# Editorial: The Vascular Niche in Tissue Repair: A Therapeutic Target for Regeneration

**DOI:** 10.3389/fcell.2017.00088

**Published:** 2017-09-29

**Authors:** Francisco J. Rivera, Maria Elena Silva, Ludwig Aigner

**Affiliations:** ^1^Laboratory of Stem Cells and Neuroregeneration, Institute of Anatomy, Histology and Pathology, Faculty of Medicine, Universidad Austral de Chile, Valdivia, Chile; ^2^Center for Interdisciplinary Studies on the Nervous System (CISNe), Universidad Austral de Chile, Valdivia, Chile; ^3^Institute of Molecular Regenerative Medicine, Paracelsus Medical University, Salzburg, Austria; ^4^Spinal Cord Injury and Tissue Regeneration Center Salzburg (SCI-TReCS), Paracelsus Medical University, Salzburg, Austria; ^5^Institute of Pharmacy, Faculty of Sciences, Universidad Austral de Chile, Valdivia, Chile; ^6^Austrian Cluster for Tissue Regeneration, Vienna, Austria

**Keywords:** vascular niche, tissue repair, regeneration, angiogenesis, stem cells, endothelial cells, pericytes, platelets

In mammals, although regeneration is quite restricted to a number of tissues and organs, this particular healing process is possible through the existence of tissue-resident stem/progenitor cells. Upon injury, these cells are activated, they proliferate, migrate, and differentiate into tissue-specific cells and functionally replace the damaged or lost cells. Besides this, angiogenesis and neovascularization play crucial roles in tissue repair. Blood vessels (BV) together with the resident surrounding cells create a vascular niche which is central to local and distant signaling thereby shaping the regenerative response.

The Frontiers Research Topic “The Vascular Niche in Tissue Repair: A Therapeutic Target for Regeneration” encompasses 14 articles highlighting various aspects of the vascular niche (VN) in health and disease. This research topic first describes *ex vivo* methodological aspects to study the role of the VN in regeneration, second addresses the VN cellular composition and roles during regeneration, third described local as well as distant signaling mechanisms regulating the VN in regeneration and, finally addresses the VN responses in pathology.

## Vascular niche: methodological insights in the study of angiogenesis and regeneration

Two papers highlight special methods to study the VN. Hutter-Schmid et al. present organotypic brain slices cultures as a tool and a system to study angiogenesis in the brain. This is certainly a very valuable method as it presents cells in their physiological context and provides a screening tool for the search for factors to modulate angiogenesis and neovascularization in the brain.

Chavez et al. present the zebrafish as an organism to study angiogenesis in development and regeneration *in vivo*. In addition, this review summarizes also other currently available *in vitro* and *in vivo* experimental models to study angiogenesis.

## Vascular niche: cellular composition and roles in regeneration

Over the past years, the view of the vascular system as a sole provider for oxygen and nutrients has dramatically changed. First, endothelial cells, which form the inner layer of the blood vessel wall, are in direct contact at the luminal face with blood-derived elements and on the outer face with other various parenchymal cell types facilitating the communication between all these components, creating a functional VN.

Koutsakis and Kazanis highlight in their article the role of the vascular system in neural stem cell (NSCs) biology and neurogenesis mainly from an evolutionary and developmental point of view. During early development, the generation and specification of NSCs apparently does not require the presence of BV, as specification of neuroectoderm and of neuroepithelial cells precedes angiogenesis. In contrast, in the adult central nervous system (CNS), NSCs are in direct contact or at least in close proximity to BV, allowing for a direct interaction between these two cell types. Even though signals derived from endothelial cells can modulate NSC proliferation and differentiation *in vitro*, BV mainly influence and support migration of neuronal precursors and young immature neurons toward their final destination. The concept addressed by these authors is well supported by the fact that BV serve endogenous oligodendrocyte progenitor cells (OPCs) as scaffolds for migration during myelination (Tsai et al., 2016). This, of course, may provide opportunities for the future design of approaches to target progenitors to specific areas of need.

Besides endothelial cells, pericytes have recently moved into the focus of attention as an essential player for different roles of the VN. Trost et al. review the current knowledge on brain and retinal pericytes. Classically, pericytes contribute to vascular homeostasis such as vessel stabilization, blood flow regulation, and the formation of the blood brain barrier. However, a number of studies have shown that pericytes display unexpected functions, beyond vascular homeostasis, as this cell type supports tissue repair and regeneration. Indeed, pericytes are multipotent (Crisan et al., 2008) being able to give rise to cells that form the fibrotic scar after acute injuries (Goritz et al., 2011). In addition, pericytes may act as stromal cells and modulate the function of neighboring local stem and progenitor cells in their regenerative activities. Supporting this concept exposed by Trost et al., we have recently shown that in response to demyelination pericytes modulate CNS-resident OPC function during myelin regeneration (De La Fuente et al., 2017). The different modes of action of pericytes might be explained by the existence of heterogeneous subpopulations with different functions, as their embryonic origin can either be neuroectodermal/neural crest or mesodermal. However, this issue needs further investigation. Finally, considering the similarities between pericytes and mesenchymal stem cells (MSCs) Schimke et al. review the clinical potential of MSCs-based therapies to favor tissue regeneration and in which extend aging might affect this therapeutic feature. The review stretches from cell phenotyping to cell therapy concepts.

Traditionally, the vasculature and the ventricular/spinal canal have been considered to be the main fluid systems in the CNS for supply and for drainage. Here, Guerra et al. highlight the importance of the subcommisural organ and other periventricular secretory structures in regulating neurogenesis. These structures contribute to the cerebrospinal fluid, which is essential in the regulation of NSC activities. More recently, it has been revealed that the brain and the spinal cord do have also a lymphatic system. In line with the previous, Kaser-Eichberger et al. provide a descriptive immunohistological study on the lymphatic system with a focus on the spinal cord. The lymphatic system in the CNS needs to be explored more in detail, as it provides a mechanism for clearance of substances and any malfunction might lead to detrimental consequences for the brain.

Another entity of the VN in the CNS seems to be the platelets. While the main function of platelets is to control blood clotting and thereby repair of vascular damage, more recent data demonstrate that platelets might be involved in the regulation of tissue repair and regeneration. Rivera et al. point out in an opinion paper that platelets are involved in neuroinflammation and hence might promote degenerative processes in the CNS. On the other hand, they might as well be beneficial as they promote regeneration through acting on endogenous progenitors. Besides their effects on neural progenitors, platelets and platelet derived factors affect angiogenesis, this particular topic is illustrated in the review article by Martinez et al. Nevertheless, the contribution of platelets in tissue repair and regeneration is quite unexplored.

In huge contrast to most regenerative niches of the body, the tendon has very little vasculature. Tempfer and Traweger describe the tendons as an atypical niche since they are almost avascular and angiogenesis and neovascularization seem to hamper their regeneration. Therefore, this review article opens the idea that depending on the tissue, one might either use pro- or anti-vascularization approaches to promote regeneration.

## Vascular niche: molecular signaling in regeneration

Blood circulating factors modulate tissue regeneration through the VN. This enables local as well as distant signaling. Regenerative soluble factors can either be produced locally or at distal sites reaching the target tissue through the blood stream. Similarly, cells produced at distant locations might reach the target tissue through the vasculature, and might then modulate local regeneration. Wallner et al. review on the role of granulocyte colony stimulating factor (G-CSF) in angiogenesis and neuroregeneration upon spinal cord injury and during Amyotrophic Lateral Sclerosis. G-CSF is a multimodal and pleiotropic factor modulating a wide spectrum of events including neurogenesis and angiogenesis, eventually, boosting tissue regeneration leading to an improvement of neurological function in patients.

Besides the classical growth factor/cytokine mediated signaling, communication via extracellular vesicles/exosomes has recently moved into the center of interest. Batiz et al. provide a comprehensive overview on: (i) the adult neurogenic niches and their cellular components; (ii) classical signaling mechanisms regulating the neurogenic niches such as neurotransmitters, hormones, cerebrospinal fluid-derived as well as blood-derived factors, and; (iii) exosome mediated signaling at the neurogenic niche.

## Vascular niche: regeneration in disease

The VN is massively altered in diseases and often, vascular changes are crucial in the process of pathogenesis. Fierro et al. present novel data on how hypoxic pre-conditioning leads to an increased infiltration of endothelial cells into scaffolds for dermal regeneration. Here, MSCs were seeded in scaffolds and angiogenic features *in vitro* and after implantation *in vivo* were studied. This is of relevance in particular for the field of biomaterials and tissue engineering for repair and regeneration.

Original data were also presented by Klein et al., who describe how microglia-mediated neuroinflammation induced by allergic conditions impact in adult hippocampal neurogenesis. Very surprisingly, acute allergic reactions in the periphery caused an elevation of neurogenesis.

## Author contributions

FR and LA conceived the idea and prepared the manuscript. MS designed, corrected and edited the manuscript.

### Conflict of interest statement

The authors declare that the research was conducted in the absence of any commercial or financial relationships that could be construed as a potential conflict of interest.
